# Protective Vaccination against Infectious Bursal Disease Virus with Whole Recombinant *Kluyveromyces lactis* Yeast Expressing the Viral VP2 Subunit

**DOI:** 10.1371/journal.pone.0042870

**Published:** 2012-09-14

**Authors:** Marina Arnold, Vijay Durairaj, Egbert Mundt, Katja Schulze, Karin D. Breunig, Sven-Erik Behrens

**Affiliations:** 1 Institute of Biochemistry and Biotechnology, Faculty of Life Sciences (NFI), Martin Luther University Halle-Wittenberg, Halle, Germany; 2 Institute of Biology, Faculty of Life Sciences (NFI), Martin Luther University Halle-Wittenberg, Halle, Germany; 3 Poultry Diagnostic and Research Center, College of Veterinary Medicine, The University of Georgia, Athens, Georgia, United States of America; 4 IDT Biologika GmbH, Dessau-Rosslau, Germany; Hallym University, Republic of Korea

## Abstract

Here we report on vaccination approaches against infectious bursal disease (IBD) of poultry that were performed with complete yeast of the species *Kluyveromyces lactis* (*K. lactis*). Employing a genetic system that enables the rapid production of stably transfected recombinant *K. lactis*, we generated yeast strains that expressed defined quantities of the virus capsid forming protein VP2 of infectious bursal disease virus (IBDV). Both, subcutaneous as well as oral vaccination regiments with the heat-inactivated but otherwise untreated yeast induced IBDV-neutralizing antibodies in mice and chickens. A full protection against a subsequent IBDV infection was achieved by subcutaneous inoculation of only milligram amounts of yeast per chicken. Oral vaccination also generated protection: while mortality was observed in control animals after virus challenge, none of the vaccinees died and ca. one-tenth were protected as indicated by the absence of lesions in the bursa of Fabricius. Recombinant *K. lactis* was thus indicated as a potent tool for the induction of a protective immune response by different applications. Subcutaneously applied *K. lactis* that expresses the IBDV VP2 was shown to function as an efficacious anti-IBD subunit vaccine.

## Introduction

Infectious bursal disease virus (IBDV) is the causative agent of infectious bursal disease (IBD), a highly contagious immunosuppressive disease in young chickens. IBDV is a non-enveloped virus; it consists of the bi-segmented double-strand RNA genome and the genome-enclosing viral capsid that is mainly formed by the viral protein VP2 [1], [2]. IBDV infects premature B-lymphocytes [3]–[5], and the primary effect of IBD is caused by the depletion of B-lymphocytes [6] that impairs the animal’s ability to develop antibodies [7]. In the poultry industry, IBD is controlled by administration of live, inactivated or recombinant IBDV vaccines [8]–[13]. Repeated vaccination of breeder hens leads to an enduring high serum antibody response [14], and progeny chickens receive maternal-derived antibodies (MDA) *via* the yolk sac that provide protection for the first few weeks after hatching [15]. The appearance of IBDV variants in the US [16], [17] and very virulent strains in Europe [18], [19] that may escape from MDA resulted in recent changes of vaccination regimes with broilers getting vaccinated with more virulent vaccines at 2–3 weeks of age when the MDA have declined [20], [21].

The presently used inactivated vaccines are effective against IBD but include certain disadvantages. While the obtained titers of antibody may not considerably vary from hen to hen in one flock of similar age, the offspring of various vaccinated flocks may show different IBDV antibody titers. When raised together, this may result in different levels of MDA in the offspring and compartmentalizes the herd into individuals with low or high susceptibility to virulent IBDV. Moreover, due to a poor efficiency, the available inactivated vaccines need to be applied at least twice, which is labor intensive and costly. Live attenuated vaccines entail the risk of not taking as they may be neutralized by the MDA. To overcome the latter problem, more virulent live vaccines have been used in the field, which may cause bursal damage themselves [22]. Accordingly, there is a need to develop new vaccines that combine a straight forward administration with high efficacy and few side-effects. An attractive approach involves the development of effective subunit-based vaccines as this would eliminate the use of embryonated eggs or live birds for bursal vaccine production. In addition, the use of material that exhibits a high level of biosafety and a low environmental risk would be of great benefit.

Along this line, the IBDV VP2 is in the focus of interest because this viral protein folds epitopes that induce protective immunity. That is, neutralizing antibodies against VP2 mediate a complete protection against lethal IBDV challenge [23]–[27].

Various heterologous viral vectors expressing VP2 were successfully tested in vaccination approaches [28]–[38]. Also the individual VP2 protein was shown to be highly immunogenic and protective when expressed in and purified from *Escherichia coli* [39], *Arabidopsis thaliana* [40] or from the yeasts *Saccharomyces cerevisiae* (*S. cerevisiae*) or *Pichia pastoris* (*P. pastoris*) [41]–[43]. Still, all these approaches involve either elaborate, longsome procedures to recover and grow recombinant viruses or plants, and/or expensive biochemical procedures to purify the recombinant VP2 or VP2 composed virus-like particles (vlps).

Here we describe vaccination procedures against IBD that base on fast generable recombinants of the yeast species *Kluyveromyces lactis* (*K. lactis*). Importantly, inoculation is performed directly with the complete heat-inactivated yeast.

## Results

### Generation and Optimization of *K. lactis* Strains Expressing IBDV VP2

**Figure 1:**
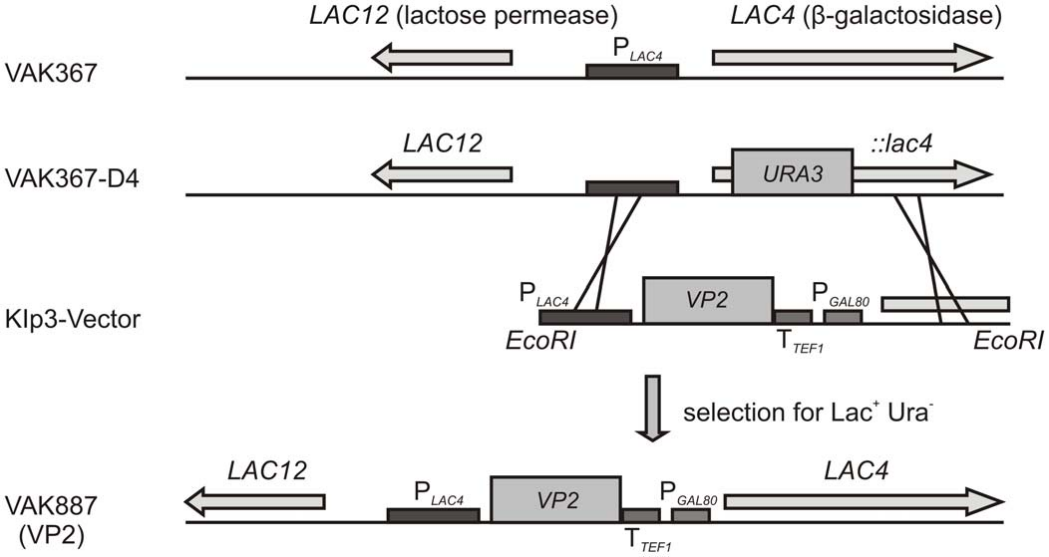
Generation of recombinant *K. lactis*. Upper panel: Schematic representation of the genomic organization of the *LAC* genes of *K. lactis* strain VAK367. The localization of the *LAC4* and *LAC12* genes and the directions of transcription are indicated by arrows. Lower panel: Schematic representation of the genomic organization of the mutated *LAC4* gene of *K. lactis* strain VAK367-D4 and of the recombination procedure. As described in the text, in the genome of VAK367-D4, a portion of the *LAC4* gene (indicated as *:lac4*) was substituted by the *URA3* gene (boxed). Homologous recombination with the plasmid Klp3 replaces the *URA3* gene by the gene of choice (boxed), the latter which is under control of the P*LAC4* promoter and the *TEF1* terminator. The recombination leads to the restoration of the *LAC4* ORF under the control of the P*GAL80* promoter. Selection of recombinant clones was performed on Lac+ Ura – medium.

Following the rationale of this study to inoculate animals against IBD with entire, non-fractionated yeast material, it was of central importance to generate stable *K. lactis* strains that expressed the IBDV VP2 antigen at high and reproducible amounts and to use a procedure that avoided antibiotic resistance genes for selection. For this purpose, we took advantage of a recently developed *K. lactis* variant, VAK367-D4, which permits the defined chromosomal integration of a foreign gene in a one-step procedure based on selection of transformants for growth on lactose [44]. In VAK367-D4, the 5′ end of the *K. lactis* specific *LAC4* open reading frame (ORF) is replaced by the *S. cerevisiae URA3* gene. *LAC4* encodes β-galactosidase that permits *K. lactis* to use lactose as carbon source; *URA3* encodes orotidine-5′-phosphate decarboxylase (OMP decase) that is essential for the synthesis of uracil. For the integration of a foreign gene, VAK367-D4 is transformed with a plasmid that contains an expression cassette where the gene of choice is 5′-flanked by the 5′UTR and upstream region of *LAC4* including the strong *LAC4* promoter, and 3′-flanked by the *TEF1* terminator, the *KlGAL80* promoter and the 5′-region of the *LAC4* ORF (see scheme in Fig. 1). Following homologous recombination, the *LAC4* ORF is restored and the *URA3* gene is lost. Thus, foreign gene expression is mediated by the *LAC4* promoter while *LAC4* expression is driven by the *KlGAL80* promoter. Importantly, the *LAC4* and *KlGAL80* promoters are co-regulated by the transcription activator KlGal4 (Lac9; [45]) and inducible by lactose or galactose in the yeast’s growth medium. Accordingly, the desired recombinants can be selected on lactose plates and screened for the loss of the *URA3* gene (uracil auxotrophy) and foreign gene expression is inducible by shifting glucose grown cultures to lactose containing media. The expression and activity of β-galactosidase may serve as additional indicators of gene-induction (Fig. 1).

**Figure 2:**
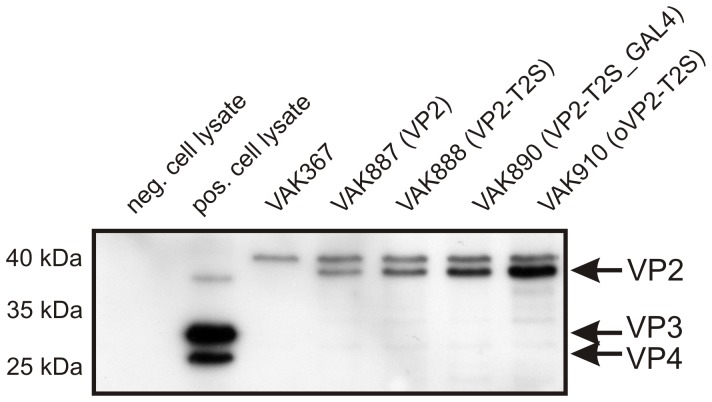
Characterization of VP2-expressing *K. lactis* strains. Total protein of extracts of naïve and IBDV-infected chicken fibroblasts (negative and positive controls) and of the various *K. lactis* strains that were generated in the course of this study (described in the text) were analyzed by SDS PAGE and Western blot using an anti IBDV antiserum. The positions of the different IBDV VP proteins in the gel are marked by arrows. As revealed by the negative control performed with protein of strain VAK367, the antiserum also reacted non-specifically with a yeast protein that migrated shortly above the heterologously expressed VP2 protein. Note that the yeast-expressed VP2 protein contained fifteen additional amino acids, which explains its slightly different migration behavior in the SDS-PAGE.

Using this system, the VP2-encoding genetic unit of the IBDV strain D78 was inserted into the *K. lactis LAC4* locus. The correct orientation of the insert was confirmed by PCR, and gene expression initially determined by measuring VP2 mRNA synthesis *via* Northern blot (not shown). Western blot analysis of yeast cell lysates further confirmed the expression of VP2 though our initially generated strain VAK887 (VP2) produced only moderate amounts of the viral protein (Fig. 2). However, in analogy to earlier findings of Jagadish *et al*. (1991) [46] in *S. cerevisiae*, VP2 protein expression could be markedly improved in strain VAK888 (VP2-T2S), where threonine at amino acid position 2 of VP2 was exchanged by serine resulting in increased protein stability (Fig. 2). A further significant optimization was achieved with strain VAK890 (VP2-T2S_GAL4). This strain contained an additional tandem integration of the *KlGAL4* trans-activator gene, which resulted in increased transcription rates of the VP2 gene (Fig. 2; transcription data not shown; details of the procedure are described by Krijger *et al*. [44]). The integration of additional *KlGAL4* genes also correlated with a higher growth rate of this strain (not shown).

### Production of VP2-expressing *K. lactis* for Subcutaneous and Oral Applications in Mice and Chickens

For the intended vaccination experiments it was important to establish protocols that enabled high density fermentation of yeast cells with high expression levels of VP2. For this purpose, the growth conditions were optimized in a bioreactor by modifying additives, pH, and an applied fed batch feeding scheme. Thus, besides increasing the amount of biomass significantly (up to 7 times in comparison to conventional flask cultures) also higher expression levels of VP2 were achieved. The applied fermentation protocol for strain VAK890 is provided as supporting information (Table S1).

After production, the yeast cells were freeze dried and heat-inactivated by incubation for 2 hrs at 90°C. For the subcutaneous immunization, the dried and powdered yeast (100 µg per vaccination in mice; 1 mg per vaccination in chickens) was mixed and applied with adjuvant. For the oral inoculation of mice, dried yeast nuggets were directly mixed with the feed resulting in a 5% (w/w) end-concentration of the recombinant, inactivated yeast. For the oral vaccination of chickens we generated and applied pellets that consisted of chicken feed and 5% (w/w) of the recombinant, inactivated yeast (see Materials and methods and below for further details).

**Figure 3:**
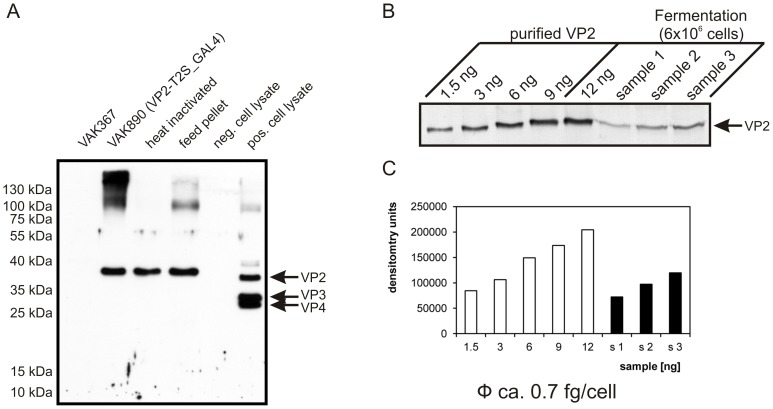
Heterologously expressed VP2 remains stable after heat-treatment and conversion of yeast into feed pellets. VP2 is expressed at similar rates in high density fermentations. (A) Western blot analysis of total protein of VAK890 yeast after heat inactivation and after encapsulation (5% w/w) into feed pellets. Comparable amounts of yeast protein were tested for the presence of VP2 using the same procedures and controls as described in Fig. 2. The VP2 protein is indicated. (B) Upper panel: Western blot of defined (ng) amounts of purified VP2. The applied amounts of purified VP2 were estimated in comparison with a standard (data not shown). The blot of purified VP2 was performed side-by-side with protein of total lysates of ca. 6×10^6^ cells (determined by platting and colony counting) of 3 independent fermentations (samples 1–3) of *K. lactis* strain VAK890. Lower panel: Densitometric measurements of the VP2 bands. The average amount of VP2 protein was calculated to correspond to ca. 0.7 ng of VP2 protein per cell.

PAGE analysis of the protein content of the heat-inactivated and feed-supplemented yeast revealed that the heterologously expressed VP2 protein stayed intact and that also its concentration per total amount of yeast protein remained constant throughout the heat-treatment and pelleting procedures (Fig. 3A). With strain VAK890, which was used in all subsequent inoculation experiments, a semi-quantitative calculation of the intracellular VP2 level indicated a concentration of ca. 0.7 fg of heterologous protein/per yeast cell (Fig. 3B).

Accordingly, in the applied immunization schemes with mice, we estimated the total amount of VP2 that was applied per subcutaneous vaccination to approximately 18 ng, while a total of approximately 340 µg of VP2 was used per oral immunization and animal. With the chickens, approximately 180 ng of VP2 was applied during subcutaneous immunization, while an amount of approximately 2.7 mg of VP2 was administered per animal for oral immunization.

### Subcutaneous and Oral Immunization of Mice and Chickens with VP2-expressing *K. lactis*

**Figure 4:**
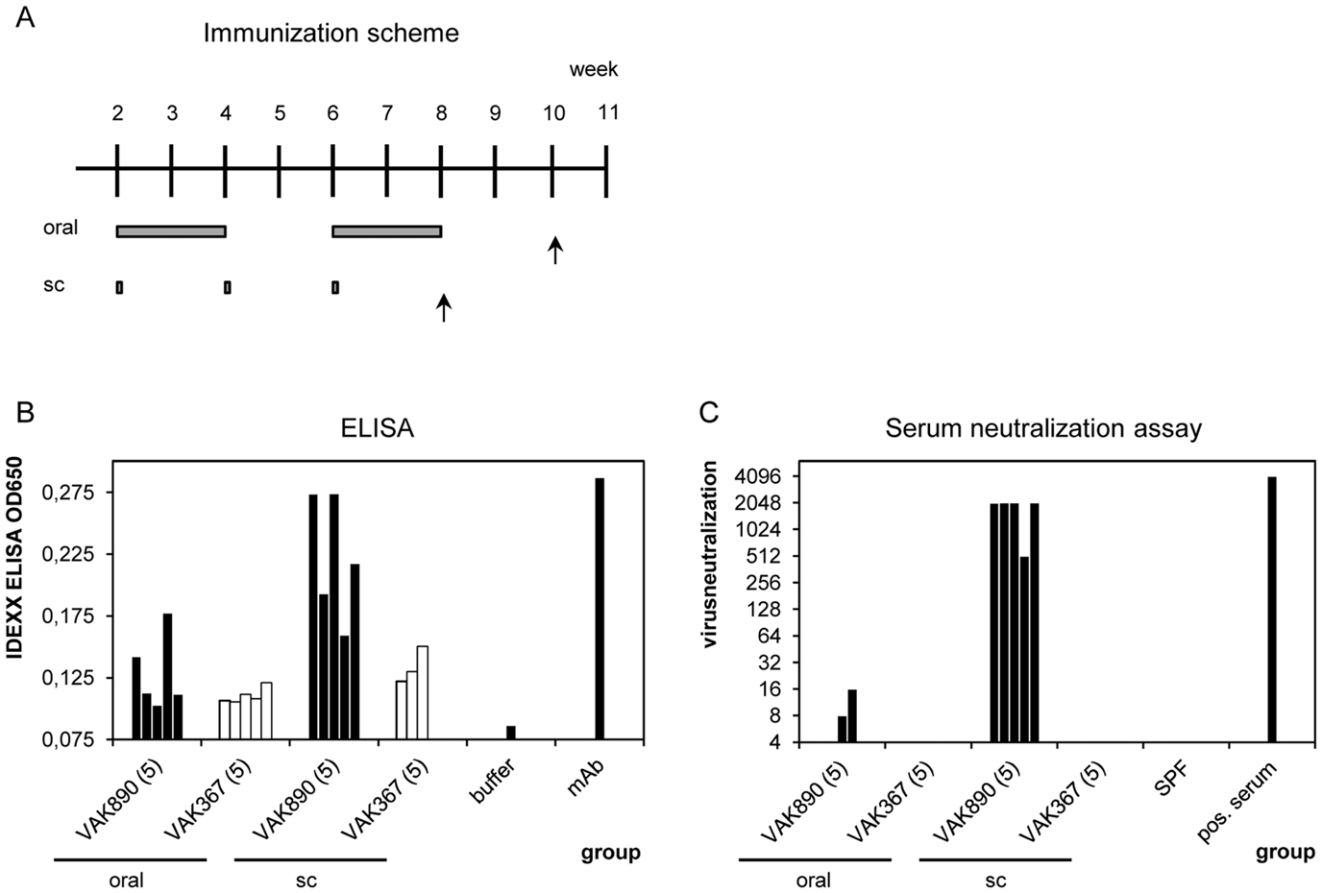
Immunization of mice with *K. lactis* strain VAK890. (A) Immunization scheme. Six weeks old mice were immunized at the indicated times/time-intervals (schematized as bars) by either subcutaneous injection (sc) or by feeding (oral) of the yeast *K. lactis* VAK890. Injection or feeding of the yeast *K. lactis* VAK367 was performed as a negative control. Arrows indicate the time points where the animals were euthanized. (B) IDEXX Elisa assay that was performed with an anti-mouse secondary antibody indicating the titers of anti IBDV antibodies in the sera of the vaccinated mice. (C) Serum neutralization (VN) assay indicating the titers of IBDV neutralizing antibodies in the sera of the vaccinated mice. A serum of a one-day-old specific-pathogen-free (SPF) chicken and a serum of an IBDV-infected chicken were used as controls.

To gain initial hints on the applicability of entire heat-inactivated yeast in vaccination approaches, we decided to test first for the induction of neutralizing antibodies against VP2 in mice. For the vaccination, we applied immunization schemes that were established during pilot-studies with other recombinant *K. lactis* strains (data not shown). Thus, within a time interval of six weeks, groups of mice were inoculated three times subcutaneously with a yeast emulsion together with a strong adjuvant or, following a 2/2/2 scheme (two weeks feeding, two weeks break, two weeks feeding), vaccinated orally with 5% yeast-supplemented feed (see Materials and methods and immunization scheme in Fig. 4A). Two weeks after the last application, the sera were analyzed for the presence of anti-VP2 antibodies with an indirect ELISA that was originally established for the detection of IBDV-specific chicken antibodies but modified such that it enabled the detection of IBDV-specific antibodies in mice. The presence of neutralizing antibodies was tested in an IBDV-neutralizing assay (VN; Fig. 4B, 4C). As summarized in Table 1, in comparison to animals that were fed with non-VP2 expressing *K. lactis* (strain VAK367), 2 of 5 mice that were orally vaccinated with VP2-expressing *K. lactis* (strain VAK890; conditions as explained) showed increased titers of IBDV specific antibodies in the ELISA and VN assay, respectively. With the subcutaneously vaccinated animals, all vaccinees revealed titers of neutralizing antibodies that were significantly higher with respect to the controls (Fig. 4, Table 1).

**Table 1:** Summary of immunization data obtained with mice.

Application	Group (No. of mice)	ELISA OD_650_	VN >2^3^
**oral**	VAK890 (5)	0.13	40%
	VAK367 (5)	0.11	0%
**sc**	VAK890 (5)	0.22	100%
	VAK367 (5)	0.13	0%

Concluding table summarizing the results of the ELISA and serum neutralization assay. Shown are the average OD values obtained with the ELISA of the whole number of mice. The dilution threshold of serum neutralization (VN) was set at 8 (2^3^) indicating that 2 of 5 mice (40%) that were orally vaccinated and 5 of 5 (100%) of the mice that were subcutaneously vaccinated with the *K. lactis* yeast VAK890 developed IBDV-specific neutralizing antibodies.

**Figure 5:**
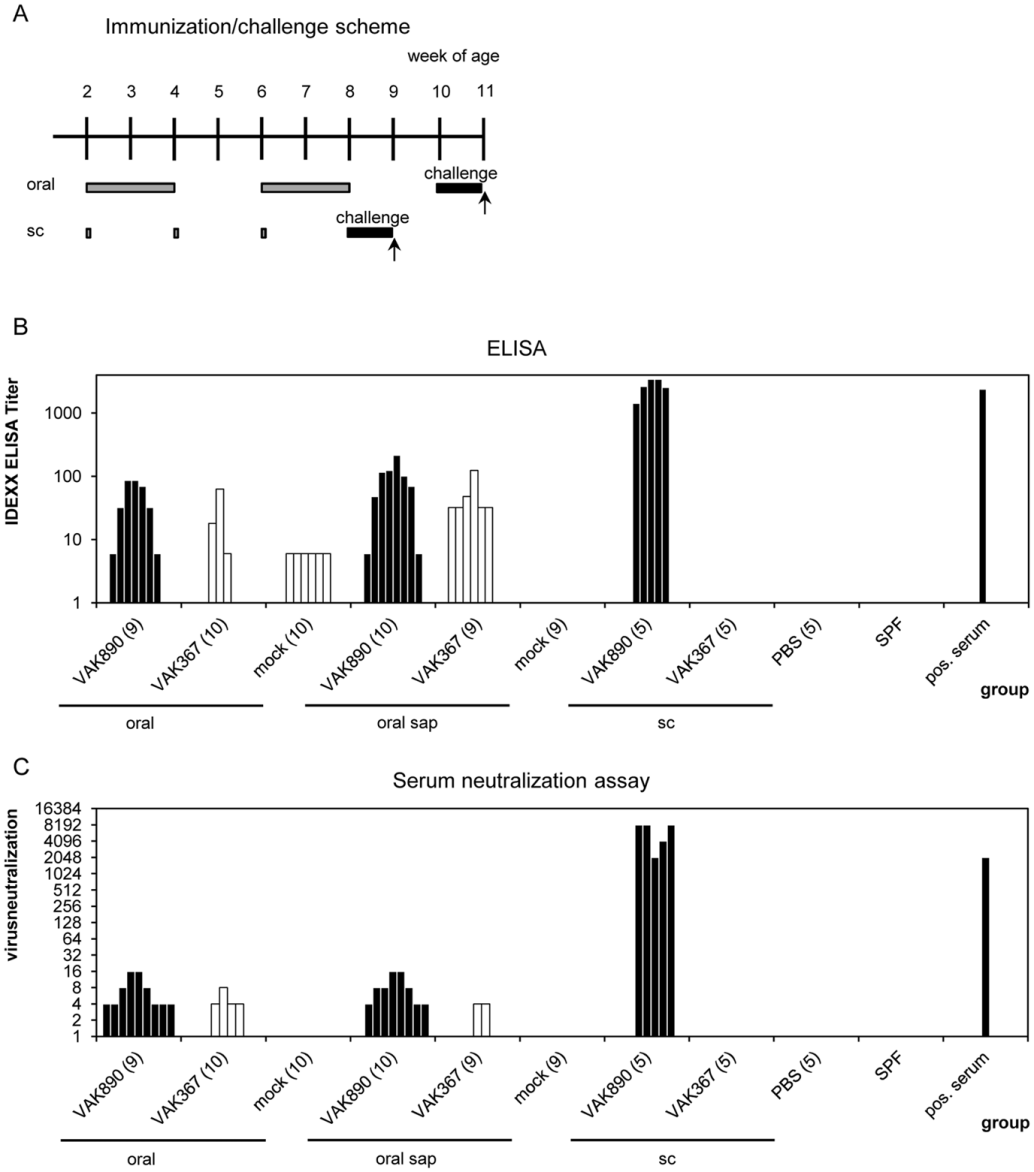
Immunization of chickens with *K. lactis* strain VAK890. (A) Immunization scheme. Two weeks old chickens were immunized at the indicated times/time-intervals (schematized as bars) by either subcutaneous injection (sc) or by feeding (oral or oral with saponin (sap)) of the yeast *K. lactis* VAK890. The yeast *K. lactis* VAK367 was applied as a negative control. The black bars indicate the time intervals that followed virus challenge; arrows indicate the time point where the animals were euthanized. (B) IDEXX Elisa assay indicating the titers of anti IBDV antibodies in the sera of the vaccinated chickens. (C) Serum neutralization (VN) assay indicating the titers of IBDV neutralizing antibodies in the sera of the vaccinated chickens. Sera of a one-day-old specific-pathogen-free (SPF) chicken, of an IBDV-infected chicken, of a mock-fed and of a phosphate-buffered saline (PBS) injected chicken were used as controls.

The apparent trend that was obtained in the experiments with mice encouraged us to next perform vaccination experiments with chickens. For this purpose, groups of leghorn chickens were immunized subcutaneously by using the same vaccination design as earlier with the mice. However, in the chickens, we used a ca. tenfold higher amount of recombinant yeast (Fig. 5A) per injection thus adapting the quantity of inoculated yeast per bodyweight to a ratio comparable with that used in mice. Alternatively, the chickens were fed with pellets that contained 5% VP2-expressing (strain VAK890) or naïve (strain VAK367) *K. lactis* applying the aforementioned 2/2/2 scheme (Fig. 5A). In addition, we performed oral vaccination with yeast after pre-treatment of the animals with saponin, an oral adjuvant that is described to generally induce Th1 and Th2 responses [47]. Importantly, after a break of one or two weeks post vaccination, all vaccinees were challenged with the classical IBDV-strain Edgar using 100 EID_50_ per chicken (Fig. 5A). This virus titer of IBDV was earlier established causing a significant IBD in the used chicken type (E. Mundt, data not shown). Employing the same assays as described above to test for serum antibody titers we obtained similar results as in the mice experiments. That is, with the oral vaccination procedure, we found that 4 of the 19 vaccinated animals displayed increased titers of virus-neutralizing antibodies. This was observed irrespective of whether the animals had been treated with saponin or not. Subcutaneous immunization with the recombinant *K. lactis* yielded high titers of neutralizing antibodies in all vaccinated animals (Fig. 5B, C). Interestingly, with both vaccination regiments, none of the vaccinees died after IBDV challenge. This was different with the control animals where the rate of mortality was 10–35% (Table 2). Determining the bursal lesion score in the collected bursae, we found that 1 bird of 19 of the orally vaccinated animals showed no signs of depletion of B lymphocytes in the bursal follicles after challenge indicating full protection against IBDV while one bird was partially protected with a bursal lesion score of 2. In the case of the subcutaneous vaccination approach, 4 of 5 animals (80%) showed a full protection while one chicken was partially protected (Table 2).

**Table 2:** Summary of immunization data obtained with chickens.

Application	Group (No. of chickens)	ELISA titer	VN >2^3^	Mortality	Lesion score
**oral**	VAK890 (9)	35.56	44%	0/9	1/4/4/4/4/4/4/4/4
	VAK367 (10)	9.4	10%	1/10	4/4/4/4/4/4/4/4/4/4
	mock (10)	4	0%	0/10	4/4/4/4/4/4/4/4/4/4
	VAK890 Sap (10)	68.8	50%	0/10	2/4/4/4/4/4/4/4/4
	VAK367 Sap (9)	33.7	0%	1/9	4/4/4/4/4/4/4/4/4
	mock Sap (9)	1	0%	0/9	4/4/4/4/4/4/4/4/4
**sc**	VAK890 (5)	2707	100%	0/5	1/1/1/1/3
	VAK367 (5)	1	0%	1/5	4/4/4/4/4
	PBS (5)	1	0%	1/5	4/4/4/4/4

Concluding table summarizing the results of the Elisa, serum neutralization assay, mortality rate and bursal lesion scores. The titers determined according to the IDEXX-instructions are given. The dilution threshold of serum neutralization (VN) was set at 8 (2^3^) indicating that 4 of 19 chickens (ca. 40%) that were orally vaccinated and 5 of 5 (100%) chickens that were subcutaneously vaccinated with the *K. lactis* yeast VAK890 developed IBDV-specific neutralizing antibodies. As indicated by lesion scores of 1 and 2, 2 of 19 chickens (ca. 10%) that were orally vaccinated and 4 of 5 chickens (80%) that were subcutaneously vaccinated with the *K. lactis* yeast VAK890 were fully protected against a subsequent IBDV infection.

## Discussion

Yeast cells are capable of expressing large quantities of foreign proteins at a relatively low cost. Accordingly, for decades *S. cerevisiae, P. pastoris* and *K. lactis* are in use as eukaryotic protein expression systems [48], [49]. More recently, particularly *S. cerevisiae* (“bakers yeast”) has been also adopted as an antigen delivery vehicle for vaccination. When subcutaneously injected, whole antigen-expressing *S. cerevisiae* was shown to activate Dendritic cells, to elicit antigen-specific cytotoxic T lymphocytes (CTL) responses and to confer protective cell-mediated immunity against tumor challenge in mice [50], [51]. Recombinant *S. cerevisiae* was also implicated as a tracer for the oral application of vaccines and drugs because the yeast cells are comparably stable, noninvasive and nonpathogenic, and they are believed to be well absorbed by M-cells in the Peyer’s patches of the gut [52]–[55].

In this study, we applied recombinants of the “milk yeast” *K. lactis* in both, subcutaneous and oral vaccination approaches. As *S. cerevisiae*, also *K. lactis* is ‘generally regarded as safe’ (“GRAS status”) for animal and human consumption [56]. However, both yeasts differ in certain physiological properties that considerably affect their usability. Thus, *K. lactis* and *S. cerevisiae* display significant differences in protein glycosylation, cell wall biosynthesis and protein secretion (reviewed by Backhaus *et al*., 2011 [57]), which may have important implications for the intracellular localization, folding, stability and immunogenicity of heterologously expressed proteins [58].

In biotechnological applications, *K. lactis* has evident advantages versus *S. cerevisiae*. Thus, glucose repression of respiratory genes and ethanol formation (“Crabtree effect”) are less dominant in *K. lactis* under aerobic conditions, which enables higher biomass yields during fermentation at high growth rates [59]–[61]. The here applied strain VAK367-D4 is particularly advantageous for fermentation purposes: as its parent strain VAK367, which was generated by mutagenesis [44], VAK367-D4 and derivatives hereof show no significant cell lysis during high-density fermentation. Moreover, *K. lactis* is one of the few yeast species that can utilize lactose, a cheap and abundant sugar, as a sole source of carbon energy. Thus, VP2 expression was controlled *via* the *LAC4* promoter and could be effectively induced by the addition of lactose to glucose grown cultures (Fig. 1 and 2).

As demonstrated here for the first time, *K. lactis* not only represents a useful system for the production and preparation of foreign proteins but also is a valuable vehicle for vaccination. That is, entire heat-inactivated material of a *K. lactis* strain (VAK890) where heterologous expression of the IBDV VP2 was improved by moderate overproduction of the KlGal4 transactivator [62], [44] (Fig. 2), induced immune responses against IBDV, the trends which were comparable in mice and chickens (Fig. 4 and 5; Tables 1 and 2). As outlined, in chickens, this immune response was at ca. 80% protective against IBDV infections in subcutaneous and partially (at ca. 10%) protective in oral approaches (Table 2); further investigations to determine if the birds without disease were still able to be infected were not conducted. Thus, within these limits and as a main result of this report, the subcutaneously applied VP2-expressing *K. lactis* turned out to be an adequate and effective anti-IBD subunit vaccine. Importantly, subcutaneous vaccination with *K. lactis* and a strong adjuvant produced a humoral response with high titers of anti VP2 and IBDV-neutralizing antibodies. In keeping with the current knowledge, we believe that these antibodies represent the major functional element of the protective immune response against IBD. In this respect our data markedly differ from subcutaneous vaccination regiments with recombinant *S. cerevisiae* that induced primarily T-cell responses [50], [63]. Hence, an important future project involves a detailed comparison of the immune responses induced by oral and subcutaneous applications of whole recombinant *K. lactis* and *S. cerevisiae*, respectively.

Another aspect worth emphasizing concerns the surprising small quantities of VP2 that turned out to be sufficient for subcutaneous vaccination with *K. lactis* against IBD. Following our estimations of the amounts of protein per yeast cell (Fig. 3), full protection of an individual chicken was achieved with approximately 180 ng of heterologously expressed VP2 only. This amount of antigen may be even further reduced since our subcutaneous vaccination scheme involved three applications of yeast; however, similar titers of neutralizing antibodies were already detectable after two applications (data provided as supporting information, Fig. S1). Considering that comparable experiments with partially purified and intramuscularly injected VP2 from *P. pastoris* applied ca. 100 µg of antigen per chicken for protective vaccination [41], it may be speculated that the adjuvant character of *K. lactis* itself plays a major role in mediating this remarkable vaccination efficiency.

Since injections are laborious, time-consuming, cause discomfort to the vaccinee and may lead to inflammations at the injection sites [64]–[68], oral applications are considered the “silver bullet” of vaccination, particularly against fecal-orally transmitted pathogens as IBDV. In mice, *K. lactis* was earlier observed to function as an oral immunogen itself (data not shown), which was explained by the high content of immunogenic β-glucans in the yeast’s cell wall and by a lack of immunotolerance to milk yeast ingredients [69]. Our data, in particular the finding that all vaccinees survived challenge and showed no clinical signs as ruffled feathers, lassitude and prostration, suggest that also oral vaccination with recombinant *K. lactis* is a promising option. However, it is obvious that a protection rate of 10% (as indicated by the lack of B-cell depletion after IBDV challenge) requires further improvement. A major issue here may concern the concentration of applied VP2. Thus, studies with transgenic rice seeds, the only example where oral vaccination of chickens against IBD turned out to be fully protective [40] applied ca. 5–10 mg of VP2 per animal versus 1–3 mg in our case. Using codon–optimization, the concentration of VP2 per yeast cell could be considerably increased (strain VAK910, Fig. 2). Other ways of optimization involve the generation of native IBDV vlps to further augment the immunogenicity of VP2. For this purpose, the commonly applied protocol for the preparation of IBDV vlp [70], which generated yet neither vlps nor subviral particles (slp) but VP2 containing particles (Fig. S2), is currently adapted to a recently established procedure that enabled the generation of vlps of murine polyomavirus in *K. lactis* (Simon *et al*., manuscript submitted). Despite that saponin had no detectable effect (Fig. 5) the systematic testing of oral adjuvants represents another promising avenue to further increase the efficiency of the oral immune response.

Taken together, our study recommends the use of non-fractionated yeast, particularly of the here-established *K. lactis* system as a powerful tool for vaccination projects. This is particularly evident considering that new vaccine strains can be generated and applied within a time frame of two to three weeks.

## Materials and Methods

### Generation of Recombinant Yeast Strains

The cDNA encoding the IBDV D78 VP2 was amplified with the following primer pair on plasmid pD78A [71]: IBDV_AscI_fwd (5′-GGCGCGCCGATGACAAACCTGCAAGATC-3′) containing an *AscI* site and VP2_NotI_rev (5′-ATAAGAATGCGGCCGCTCACACAGCTATCCTCCTTATG-3′) containing a *NotI* site. The primer pair used to generate VP2-T2S was IBDV_S:T_AscI_fwd (5′-GGCGCGCCGATGTCTAACCTGCAAGATCAAACCCA-3`) and VP2_NotI_rev. After sequence confirmation, the amplified DNA fragments were cloned *via AscI* and *NotI* into the vector KIp3-MCS (detail of construction of this plasmid provided on request). Genomic integration was performed as described in the text (Fig. 1). Specifically, the integrative plasmid was cut with *EcoRI* and the digested fragments transformed into competent VAK367*-*D4 cells. The transformed cells were platted on YPD medium [72] and incubated at 30°C overnight. For the screening of positive colonies, the transformation plate was replicate-plated on SD media (0,67% yeast nitrogen base w/o amino acid supplemented amino acids and nucleotides [11.2 mg/l adenine; 14.4 mg/l tyrosine; 38.4 mg/l of uracil, histidin, tryptophan, arginine, methionine; 48 mg/l phenylalanine; 57.7 mg/l of leucine, isoleucine, valine, threonine]) containing lactose as carbon source and incubated for 2 days at 30°C. Lactose-positive clones were further analyzed as described in the text.

The genomic integration of additional *GAL4* loci was performed as described by Kuger *et al.* (1990) [62]. The codon optimization was performed with the *S. cerevisiae* algorithm (mr.gene.com) [73]. The codon-optimized DNA fragments were synthesized with 5′ *AscI* and 3′ *NotI* restriction sites (mr.gene, Regensburg, Germany) and cloned into the Klp3-MCS vector.

### Western Blot Analysis

Pellets of yeast cells or chicken fibroblasts (DF1) that had been earlier infected with IBDV strain D78 were re-suspended in B60 buffer (50 mM Hepes-KOH pH 7.3; 60 mM potassium acetate; 5 mM magnesium acetate; 0,1% Triton X100; 10% glycerol; 1 mM sodium fluoride; 20 mM glycerophosphate; 10 mM MgCl_2_; 1 mM DTT; complete protease inhibitor (1 tablet/50 ml; Roche)) and disrupted by vortexing in the presence of glass beads. The extract was centrifuged (14000 rpm, 20 min at 4°C) and the protein concentration determined by a standard procedure. 40 µg of protein extract were separated by 12% SDS-PAGE, the proteins transferred and Western blot analyses carried out with a rabbit anti-IBDV antiserum (1∶15,000; [74]) and a goat α-rabbit HRP-coupled antibody (1∶3000; Santa Cruz Biotechnology, Inc.) using standard procedures.

### Northern Blot Analysis

For total RNA extraction, yeast cells of a 5 ml culture were pelleted and immediately chilled on ice. Cell lysis was performed in ProtK Buffer (100 mM Tris/HCl pH 7.9, 150 mM NaCl, 25 mM EDTA, 1% (w/v) SDS) and 50 mg Proteinase K (Fermentas) by vigorous vortexing in the presence of glass beads. The samples were incubated for 1 h at 35°C and the RNA was extracted, precipitated and re-suspended in RNAse-free water. Northern blotting was performed as previously described by Engler-Blum *et al*. with some modifications [75]. Briefly, 5 µg of total RNA were separated on a 1% (w/v) agarose gel (containing 1.85% (v/v) formaldehyde) and the gel blotted onto a nylon membrane (Amersham HybondTM-N*+*, GE Healthcare). The membrane was hybridized at 68°C with a DIG-labeled RNA probe that had been generated by *in vitro* transcription from PCR fragments in the presence of DIG-NTPs (Roche). The blot was treated with a blocking solution and incubated with an anti-DIG alkaline-phosphatase-conjugated antibody (Roche). The detection of alkaline-phosphatase activity was performed using standard procedures.

### Concentration and Purification of Heterologously Produced VP2

For the purification of IBDV VP2, we applied the protocol of Saugar *et al*., 2005 [70] that was developed for the purification of IBDV VP2 vlps from infected chicken cells. 2000 odu (optic density units) of a yeast culture, the cells that had been transformed with an episomal VP2 plasmid (pADH1-P_VP2-T2S; construction detail provided on request), were grown on auxotrophic SD-uracil media with 2% glucose overnight. After harvesting and washing with distilled water, the cells were disrupted with glass beads in lysis buffer (10 mM Tris [pH 8.0], 150 mM NaCl, 20 mM CaCl_2_, 1 mM EDTA, complete protease inhibitor (1 tablet/50 ml; Roche). The resulting protein extract was centrifuged (10,000 x g for 1 h at 4°C) and the soluble fraction applied to a 20% (w/v) sucrose cushion in sucrose buffer (10 mM Tris pH 8.0, 150 mM NaCl, 20 mM CaCl_2_) containing complete protease inhibitor. After centrifugation at 170,000×g for 3 h at 4°C, the pellet was solved in 200 µl sucrose buffer and further centrifuged (17 h 114.000×g) on a 20–53% (w/v) sucrose gradient in sucrose buffer. The gradient was harvested in 700 µl fractions and the heterologously expressed VP2 which formed neither vlps nor slps but protein particles under the applied conditions was thus purified and concentrated. The amount of protein in the peak fraction was estimated by SDS-PAGE and Coomassie staining in comparison to a standard protein (BSA; Fig. S2) and the estimated amounts correlated with signals obtained in parallel-performed Western blots (Fig. 3).

### Yeast Production and Heat killing

All experimental fermentations and optimizations were carried out in a DasGip parallel bioreactor system (DasGip AG, Jülich, Germany) with four 2L fully equipped fermenters. Fermentations for production purposes were performed by Organobalance GmbH (Berlin, Germany) or in a Biostat ED bioreactor with a 10L working volume (B. Braun Biotech, Melsungen, Germany). All production processes were operated in a fed-batch mode. A complex culture medium with some defined additives was used; the compositions of finally used media and feed solutions will be supplied on request. The temperature of the yeast culture was maintained at 30°C and the pO_2_ controlled to 30%sat. The pH during culturing was set to 5.0 by the addition of 2M NaOH and 2M H_3_PO_4_, respectively. For the feeding experiments with mice and chickens, the yeast was freeze dried and subsequently heat-killed for 2h at 90°C. Using this procedure, less than 10 cells per gram dry cell weight remained viable.

### Vaccination of Mice

Ethics Statement I: all experiments with mice were performed along the guidelines and by approval of the Animal Care and Use Committee of the Landesverwaltungsamt Sachsen-Anhalt, Germany and in accordance with the EU Directive 2010/63/EU for animal experiments (Permit Numbers 42502-2-1055MLUÄ and 42502-2-1088MLUG). During injection and for euthanasia the mice were anesthetized with 1.5% isoflurane and all efforts were made to minimize suffering.

The immunization experiments were performed with six weeks old female mice (CL57BL/6; Harlan Laboratories, The Netherlands) in groups of five individuals. For subcutaneous immunization, the dried, powdered yeast was mixed with complete Freund’s adjuvant (CFA; Sigma) in the first application and with incomplete Freund’s adjuvant (IFA; Sigma) in the subsequent booster vaccinations. Per immunization/boost step, 200 µl emulsion (containing 100 µg of yeast) was injected per mouse. During the injection procedure (number of application as indicated in Fig. 4A), the mice were anesthetized with 1.5% isoflurane. After initial injection, the mice were boosted twice in two weeks intervals (i.e., at day 14 and 28, respectively; Fig. 4). For the oral immunization, dried yeast nuggets were mixed with mice feed to an end concentration of 5% (w/w). Each mouse was fed twice with 2 g of total feed per day. The yeast-containing feed was applied for four weeks with a two weeks break (Fig. 4A). Two weeks after the last subcutaneous or oral application, the mice were euthanized with 1.5% isoflurane and blood samples taken from vena cava.

### Vaccination of Chickens

Ethics Statement II: all studies with chickens were conducted in BSL-2 approved animal facilities at the Poultry Diagnostic and Research Center at the University of Georgia. The experiments were approved by the Institutional Animal Care and Use Committee of the University of Georgia (IACUC number A2010 12-017-Y1-A0). For euthanasia, the birds were anesthetized with CO_2_ and all efforts were made to minimize suffering.

The immunization experiments were performed with fourteen-days-old specific pathogen-free (SPF) leghorn chickens in groups of up to 10 individuals. For subcutaneous vaccination, 5 mg of dried, powdered yeast were solved in 750 µl PBS and 500 µl of sterile distilled water and an emulsion was prepared with 1.25 ml IFA. 500 µl of this emulsion (containing 1 mg of yeast or only PBS) was injected subcutaneously at day 0, 14 and 28, respectively (see Fig. 5). For oral immunization, the dried and heat-killed yeast was mixed with chicken feed and pellets with a yeast concentration of 5% (w/w) were formed. The birds were fed once a day with increasing amounts of feed depending on their age. The immunization scheme was performed as described in the text and shown in Fig. 5. The treatment with saponin (*Quillaja saponaria* Molina, Sigma) was performed prior to the initiation of the oral immunization. That is, 5 mg of saponin were dissolved in PBS containing 30 mg/ml NaHCO3, and 1 mg of saponin solution was applied per bird by esophageal intubation [76]. The animals were bled prior to each application; the last bleed was done two weeks after the last application. Using the oral route, the birds were challenged with 100 EID_50_ of the classical IBDV Edgar strain (kindly provided by Dr. Holly Sellers, University of Georgia, Athens). Seven days post challenge the birds were euthanized with CO_2_, the bursae of Fabricius were collected, fixed for 24 h in 10% neutral-buffered formalin and embedded in paraffin.

### Enzyme-linked Immunosorbent Assay (ELISA)

IBDV specific antibody titers in the chickens sera were determined with the IDEXX FlockChek® IBD ELISA kit (IDEXX Laboratories, Inc.) using the procedure recommended by the manufacturer. The same ELISA was applied to the mice sera; in the latter case, an anti-mouse HRP-coupled secondary antibody was used (Sigma Aldrich, 1∶5000). Since this ELISA was established for the detection of IBDV-specific chicken antibodies, the IBDV VP2-specifc antibody R63 was used as a positive control [77].

### Virus Neutralization Assay

The assay to determine IBDV neutralization by mice and chickens sera was performed essentially as described by Schröder *et al*., 2000 [78]. Virus strain D78 was applied. As a negative control for the virus neutralization assay, a serum pool obtained from SPF one-day old chickens (Merial, Gainesville, GA, USA) was used. The positive control serum was obtained after vaccination of ten three-week-old SPF chickens (Merial) with the live IBDV vaccine NOBILIS GUMBORO D78 (Intervet, Millsboro, DE, USA) according to the manufacturer’s recommendation. Three weeks after vaccination, the chickens were exsanguinated and the serum pool was used for the assays.

### Histopathology

Four micrometer thin tissue sections were obtained from the parafinned bursae of Fabricius. These were de-parafinned and stained with hematoxylin and eosin following standard procedures. The samples were microscopically evaluated and the lesion score [79], [80] was determined on a scale from 1 to 4 (1 = normal to 10% follicular atrophy; 2 = 10–30% follicular atrophy; 3 = 30–70% follicular atrophy; 4 = >70% atrophy). The presence of up to 10% follicular atrophy was attributed to individual apoptotic cells that affect each follicle during the normal development of the bursa. This can only be attributed to sections of the bursae of Fabricius obtained from SPF animal under controlled experimental conditions.

### Data Analysis

Mean antibody titers between groups were compared by SigmaStat® (SigmaStat for Windows, Jandel Scientific, San Rafael, CA). Differences between two groups were conducted by one-way analysis of variance followed by Fisher LSD for all pairwise multiple comparisons.

## Supporting Information

Figure S1 **Serum neutralization assay indicating the titers of IBDV neutralizing antibodies in the sera of the subcutaneously vaccinated chickens after each application.** The sera were collected 13 days after each shot (Fig. 5A). Serum of a one-day-old specific-pathogen-free (*SPF*) *chicken* and serum from an IBDV-infected chicken were used as controls.
(PPTX)

Figure S2 **Purification and semi-quantification of IBDV VP2.** Protein extracts of VAK890 and VAK367 cells were supplied to two centrifugations *via* a sucrose cushion (20%) and a subsequent sucrose gradient (20–53%) following the protocol of Saugar *et al*., 2005 [68] (A). Fractions of the sucrose gradient (F1 (20%)–F18 (53%)) were analyzed by western blot for the presence of VP2. Electron Microscopy (not shown) revealed that fractions F6–F8 contained higher-sedimenting particles of VP2 that contained the protein highly enriched. (B) The amount of VP2 contained in fraction F7 (pVP2) was compared side-by-side with standard amounts of bovine serum albumin (BSA) in a Coomassie stained SDS-PAGE using densitometry of the individual protein bands. Fraction F7 that was prepared from protein samples of strain VAK367 served as a negative control (contr.).
(PPTX)

Table S1 **Conditions under which high density fermentation of** ***K. lactis*** **strain VAK890 was performed.**
(PPTX)
